# Student Perceptions of a Virtual Reality Animation for Teaching Absorption and Bioavailability in Pharmacology: A Mixed Methods Evaluation

**DOI:** 10.1002/prp2.70294

**Published:** 2026-07-01

**Authors:** Jon Andsnes Berg, Trond Trætteberg Serkland, Monika Kvernenes, Johnson Liu, Waltraud Binder, Steve Gallagher, David Reith, Ullamari Pesonen, Dimitra Mitsa, Marit Christine Strandvik, Tiril Egset Mork, Silje Skrede, Paul White

**Affiliations:** ^1^ Department of Clinical Science University of Bergen Bergen Norway; ^2^ Section of Clinical Pharmacology, Department of Medical Biochemistry and Pharmacology Haukeland University Hospital Bergen Norway; ^3^ Center for Medical Education University of Bergen Bergen Norway; ^4^ School of Biomedical Sciences, Faculty of Medicine & Health UNSW Sydney Kensington New South Wales Australia; ^5^ Medical Education Unit, Office of the Dean, Dunedin School of Medicine University of Otago Dunedin New Zealand; ^6^ Department of Biomedicine University of Turku Turun yliopisto Finland; ^7^ School of Molecular and Cellular Biology, Faculty of Biological Sciences University of Leeds Leeds UK; ^8^ Department of Game Development University Inland Norway Elverum Norway; ^9^ Faculty of Medicine University of Bergen Bergen Norway; ^10^ Faculty of Pharmacy and Pharmaceutical Sciences Monash University Parkville Victoria Australia

**Keywords:** absorption, bioavailability, education, medication errors, virtual reality

## Abstract

Medication errors, often arising from insufficient pharmacology knowledge, can have serious consequences, highlighting the importance of effective pharmacology education for health care students. This study hypothesized that virtual reality (VR) could improve student engagement, motivation and perceived learning of core concepts in pharmacology. A mixed‐method approach was employed. Students who had completed a course in basic pharmacology were recruited from five international study sites to view a VR animation, explaining drug absorption and bioavailability. The students responded to an online questionnaire exploring their experience and understanding. In addition, 13 medical students from the University of Bergen participated in focus group interviews to further explore their perceptions of VR in pharmacology education. A total of 133 students participated in the VR session and completed the questionnaire, with approximately half reporting that the VR animation changed their understanding of drug absorption. Thematic analysis of the focus group interviews produced three themes descriptive of the students' learning experiences, each pivoting tensions between: (1) The role of VR in integrating pharmacology with other medical disciplines; (2) striking a balance between engaging and overwhelming learning experiences; (3) in‐depth learning under the weight of assessment. The technical solution appears satisfactory, and students found the 360° VR animation engaging and useful for visualizing the complex concepts of absorption and bioavailability. VR animation shows potential to enhance integration of pharmacology to other medical disciplines. However, careful design is required to support self‐paced learning and minimize cognitive overload.

## Graphical abstract

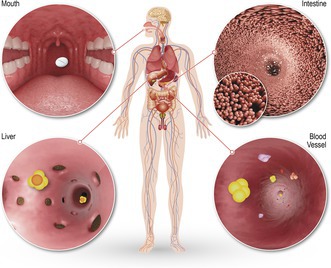

**Abbreviations**
AI: artificial intelligence
AR: augmented reality
HMD: head mounted display
VR: virtual reality

## 1 Introduction

Medication errors have significant economic consequences and contribute to avoidable patient morbidity and mortality [1]. It has been estimated that a total of 0.7% of the global health expenditure could be saved if medication errors were prevented [2]. Studies have shown that prescribing errors among recently graduated medical doctors are often due to a lack of pharmacological knowledge [3, 4, 5]. Knowledge gaps have been identified among medical and pharmacy students, as well as health care professionals involved in medication management [6, 7, 8].

Pharmacology is often cited as a challenging subject within health professions education (HPE) and is traditionally taught through lectures in a classroom setting [9, 10]. With the aim of improving pharmacology education, an international working group has recently defined foundational core concepts (CCs) in pharmacology education [11]. These are fundamental principles that students are expected to understand and apply after completing pharmacology training. This group has investigated common misconceptions related to these CCs and developed tools to assess the students' pharmacological knowledge [12, 13, 14, 15]. Others have explored areas where pharmacology students commonly struggle [16, 17, 18]. Changes in pharmacology teaching are called for to help students overcome these common misconceptions, to achieve a solid understanding of the core concepts and apply this knowledge in practice.

Technology enhanced learning is swiftly making its way into higher education [19, 20]. Although virtual reality (VR) has been employed in HPE for training in surgical, clinical and communication skills, its application in theoretical subjects like pharmacology remains limited [21]. Within the preclinical sciences, existing literature on VR has predominantly focused on anatomy education [22]. Nevertheless, growing evidence suggests that VR has the potential to enhance student learning [23, 24, 25, 26, 27]. It introduces novel learning modalities, fostering student interest, motivation and engagement. Interest is considered a key driver of motivation [28], which in turn fuels engagement and should be present before an educational activity begins [29]. Engagement, defined as the students' active participation, is considered a predictor of academic success, increased knowledge retention, and overall well‐being [30]. Task‐related emotions, including anticipation and satisfaction, play a central role in fostering students' engagement and sustaining their motivation [31]. When these emotions are activated, for instance through VR‐based immersive approaches, they could encourage ongoing involvement in learning tasks and thereby promote deeper understanding.

In pharmacology education, VR appears particularly promising for learning enhancement because of its ability to visualize abstract and otherwise “invisible” processes. It enables immersive learning experiences where students can develop spatial understanding [32, 33]. In VR, students can “accompany” the medication through the body, facilitating comprehension of the interactions between the body and exogenous molecules, manifesting within a three‐dimensional space.

We developed a pilot VR animation to explore whether VR could be implemented as a learning resource in pharmacology for HPE students. The animation is showing the absorption process of an orally administered tablet, covering the CCs of absorption and bioavailability. With the present study we aimed to evaluate how VR technology influences student motivation, engagement, and perceived learning.

Based on our findings, we also present key lessons to inform educators interested in developing VR‐based material for educational purposes.

## 2 Materials and Methods

### 2.1 Study Design

We used a sequential mixed‐methods approach to investigate students' experiences. The study comprised both a quantitative component, in which participants completed a questionnaire immediately after viewing the VR video, and a qualitative component, in which a subset of participants took part in focus group interviews.

### 2.2 Data Collection

#### 2.2.1 Study Participants

Students were recruited from medicine, biomedicine and pharmaceutical sciences programmes that included a basic pharmacology course at five universities: University of Bergen (UiB, Norway), Leeds University (UoL, United Kingdom), University of Turku (UoT, Finland), University of Otago (UoO, New Zealand) and University of New South Wales (UNSW, Australia). Due to variations in curricula and assessment across programs', the sole inclusion criterion was completion of a basic pharmacology course. Participation was voluntary, and students were recruited through in class announcements, via email invitations, through flyers, and by intranet advertisements. For the focus group interviews, conducted only at UiB, students were recruited during class sessions.

#### 2.2.2 Quantitative Data Collection

**FIGURE 1:**
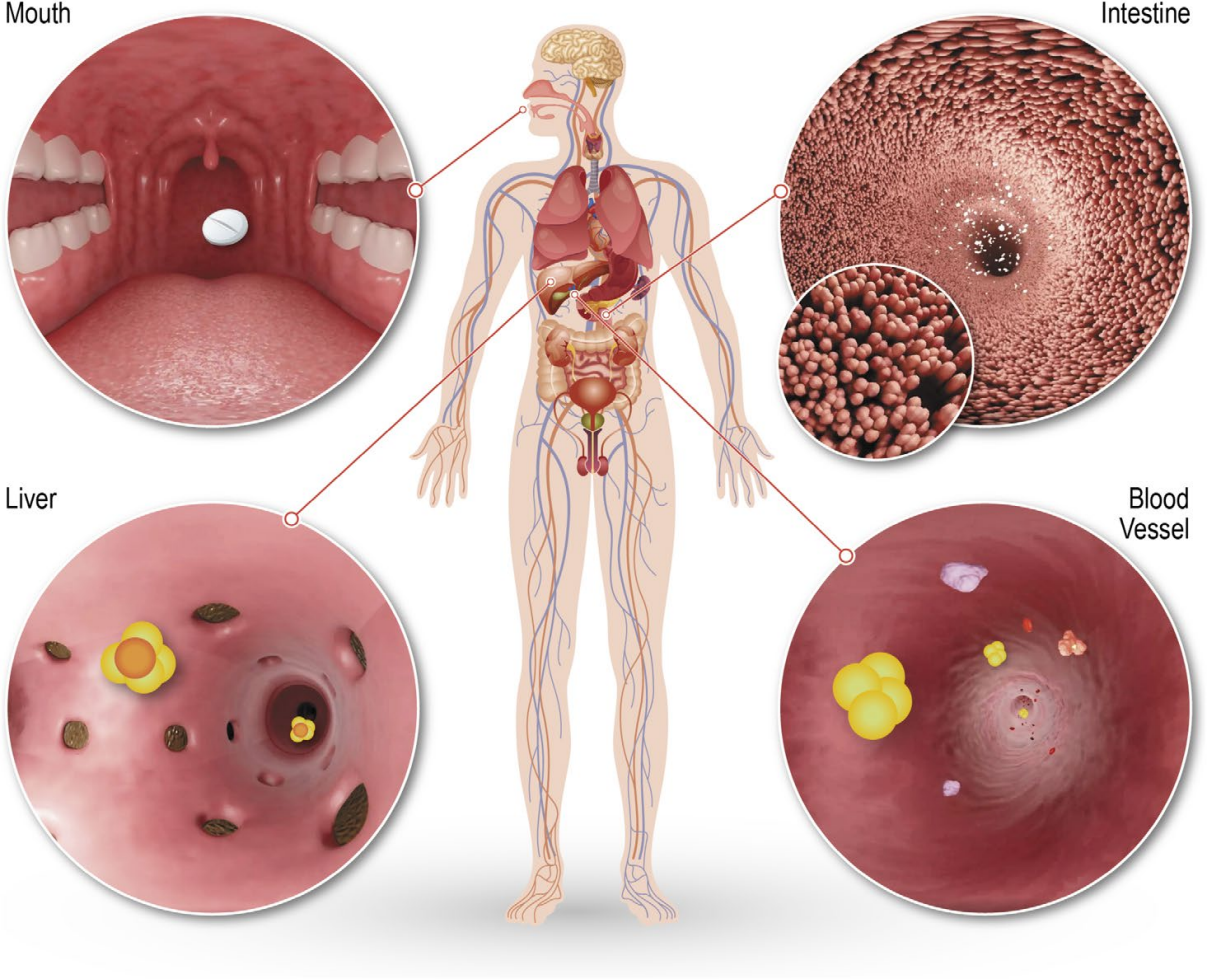
Four representative screenshots from the 360° VR animation used in the study, showing visualizations of key pharmacological processes related to oral drug absorption and bioavailability.

The VR sessions took place on campus and involved viewing a 6‐min animation illustrating drug absorption from oral administration to systemic circulation (Figure 1) [34]. Students used VR headsets provided by their respective educational institution. Different headset models were used depending on availability: Pico 2 at UiB and Meta Quest 2 and 3 at the other sites. Preliminary testing at one site indicated that upgrading from Meta Quest 1 to Meta Quest 3 headsets reduced symptoms of dizziness and nausea. The VR video featured an English voice‐over and a 360° immersive view. Users were able to pause and rewind, but no additional interactive elements were included. The video was either viewed via YouTube or preloaded onto the headsets.

Immediately after viewing the VR video, students completed an online questionnaire administered in English via SurveyXact. It took approximately 10 min to complete and included demographic questions (e.g., gender, educational background), the 10 items from the System Usability Scale (SUS), 10 statements addressing themes of motivation, engagement and understanding rated on a Likert scale, and free‐text questions designed to capture students' perceptions of the VR application (Data S1). The SUS is a validated scale, used in both academic and industry settings, to study the technical usefulness of tools or products [35]. Items relating to motivation, engagement, and perceived understanding were developed iteratively by the author group, informed by established guidance on questionnaire design in health professions education including the AMEE guidelines [36]. Data were organized and analyzed with MS Excel. Survey responses were summarized descriptively overall and by study site and program. Gender differences for the dichotomous variables were analyzed using the chi‐square test, based on complete cases only. One missing response and gender categories with very small counts were excluded. Likert‐scale responses were analyzed descriptively due to similar response patterns across gender groups. SUS scores are presented as mean values, whereas perceived changes in understanding of drug absorption are reported as counts and percentages. Given uneven and in some cases, small subgroup sizes, comparisons across study sites and programs were interpreted descriptively. Free text responses were generally brief and heterogeneous; therefore, they were not subjected to formal qualitative analysis and were used only to provide illustrative insights.

#### 2.2.3 Data Collection for the Qualitative Component

While the questionnaire captured students' ratings of motivation, engagement, and understanding of the VR animation, it did not provide in‐depth insights into their experiences. Therefore, a semi‐structured interview guide was developed in collaboration with a pedagogical researcher (M.K.), a student representative (T.E.M.), and pharmacology educators (T.T.S., J.A.B.) (Data S2). Some of the questions were created based on preliminary analysis of the questionnaire responses with particular focus on exploring students' conceptual understanding and how the VR animation supported learning. Three investigators (T.E.M., T.T.S., J.A.B.) each facilitated one focus group with medical students at UiB. From a cohort of 190 third‐year medical students, 20 were recruited. In Norway, medical education is structured as a 6‐year program that integrates both theoretical and practical training, culminating in a medical degree. A notable difference from some other countries is the early and continuous clinical exposure throughout the program, as well as the mandatory internship following graduation. Of the 20 initially recruited students, 13 ultimately participated on the scheduled interview day, forming groups of 6, 4, and 3, respectively, with a majority of participants identifying as female (*n* = 10). Some of the seven non‐participants cited conflicting educational commitments, whereas most did not provide specific reasons for their absence. The students watched the VR animation and filled out the questionnaire before the focus group interview started. The interviews lasted between 60 and 90 min and continued until no new topics or insights emerged. They were audio‐recorded and transcribed verbatim (J.A.B.) to ensure accuracy.

The data were analyzed in accordance with the principles of Braun and Clarke's reflexive thematic analysis. An inductive, interpretive approach was adopted, with analysis understood as an active and reflexive process of meaning‐making rather than a procedure aimed at coder agreement [37]. Three researchers (M.K., T.T.S., J.A.B.) were actively involved in the analysis. M.K. is an experienced qualitative researcher with a scholarly background in higher and medical education, whereas T.T.S. and J.A.B. are senior physicians and specialists in clinical pharmacology. Their extensive experience with teaching pharmacology informed the analysis and was key to recognizing issues related to students' conceptual understanding and common learning challenges. Each researcher engaged closely with the transcripts through repeated reading and coding to identify patterns and key concepts. The coded material was then reviewed and discussed among the researchers in a process involving several meetings, articulation of interpretations and refinement of suggested themes. This collaborative process led us to the identification of three main themes, which are presented in the results section. NVIVO software was used to organize and code the interview transcripts.

### 2.3 Ethics

This study was conducted in accordance with the principles outlined in the Declaration of Helsinki. Ethical approval was obtained from the Institutional Review Board (IRB) or equivalent ethics committee at each participating site. For this multisite study, all IRBs were in agreement with the study protocol and procedures. Informed consent was obtained from all participants prior to their inclusion in the study. At UoO, participating students received a voucher at a coffee shop. Participants of the focus group interviews received a voucher at the student cafeteria.

## 3 Results

### 3.1 Questionnaire

We received 133 responses with all but one questionnaire completed in full. Background data describing the questionnaire respondents is shown in Table 1.

**TABLE 1:** Descriptive data from the questionnaire, split into the participating institutions.

Descriptives	UiB	UoO	UNSW	UoT	UoL	Total
*n*	58	22	38	10	5	133
Gender						130
Woman/female	44	14	22	7	4	91
Man/male	13	7	16	2	1	39
Other/missing	1	1		1		3
Subject						
Medicine	58	22	9	3	0	92
Biomedicine			16	7		23
Pharmaceutical science			2		3	5
Other			11		2	13
Year of study						
1	0	0	0	0	4	4
2	0	0	28	3	0	31
3	27	0	8	7	0	42
4	14	22	2	0	0	38
5	17	0	0	0	1	18
Tried VR before?						
Never	30	13	15	4	1	63
One time	14	4	8	1	3	30
Two to five times	11	4	10	5	0	30
More than five times	2	1	5	0	1	9
Missing	1	0	0	0	0	1
Discomfort						
Yes	16	11	10	4	2	43
No	41	11	28	6	3	89
Missing	1	0	0	0	0	1

*Note:* Because not all participants answered every questionnaire item, the number of responses varies between variables. Missing data are shown explicitly for each item where applicable.

Abbreviations: UiB, University of Bergen; UNSW, University of New South Wales; UoL, University of Leeds; UoO, University of Otago; UoT, University of Turku; VR, virtual reality.

**FIGURE 2:**
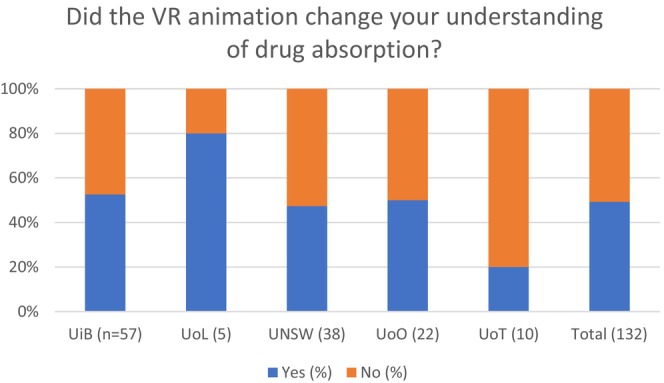
Change in understanding of drug absorption reported by the respondents. Proportion of positive and negative responses on the *y*‐axis, institutions on the *x*‐axis. UiB, University of Bergen; UNSW, University of New South Wales; UoL, University of Leeds; UoO, University of Otago; UoT, University of Turku.

Approximately half of the respondents reported a change in their understanding of absorption after viewing the VR animation (65/132, 49%; Figure 2), with no statistically significant differences observed between genders. Descriptive differences were noted across study sites, with respondents from the UoT more frequently reporting no change compared to other institutions. Across study programs, medical students most often reported a change in understanding, whereas responses from smaller program groups were more variable (Table 2). The two Likert‐scale items provided additional insight: 62% agreed or strongly agreed that the VR application made them think differently about drug absorption, whereas 78% agreed or strongly agreed that it did not conflict with their understanding (Data S3). The subgroup findings should be interpreted with caution due to small sample sizes in some categories.

**TABLE 2:** Change in understanding of drug absorption after seeing the VR animation, divided into subject groups.

Subject	Change in understanding of drug absorption		Missing
	No	Yes	
Medicine	42	49	1
Biomedicine	15	8	0
Pharmaceutical sciences	1	4	0
Other	9	4	0

*Note:* Totals may not sum to 133 because one participant did not respond to the item on change in understanding.

Free text responses further illustrated students' experiences. Several respondents reported improved understanding of the “journey” of the drug molecules from the intestines to the heart. One student wrote: “It made me realize how connected the organs are”. Some also stated that they experienced a new perspective on how a tablet dissolves in the gastrointestinal tract. Several of the students highlighted that the visualization gave them a different perspective: “It was really engaging to watch the drug move between different chambers of the body, and it was helpful in understanding the order of locations for the drug to travel to.” The immersive aspect was also emphasized: “Being immersed in the world it was easier to focus than in lectures, since all of the view was teaching.”

The students responded to Likert scale items assessing their perception of VR in pharmacology education, and in general the students' attitudes were positive (Data S3). In particular, strong agreement was observed for the statement “Using VR technology in pharmacology education can make learning more engaging,” with only three of the respondents being neutral (“neither agree, nor disagree”), or disagreeing. One hundred and twenty out of 132 completing respondents agreed or strongly agreed to the statement: “VR technology can help me understand complex pharmacological concepts more easily.” The statement “The benefits of using VR technology in my field of study do not outweigh the challenges and limitations.” collected more mixed responses. Seventy‐three students disagreed or strongly disagreed, 32 agreed or strongly agreed, and 27 neither agreed nor disagreed.

**FIGURE 3:**
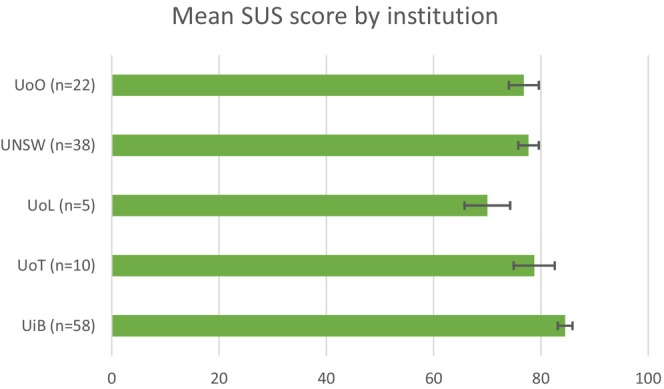
Mean system usability score (SUS) by institution. Error bars represent standard errors of the mean (SEM). Sample sizes are shown on the *y*‐axis. UiB, University of Bergen; UNSW, University of New South Wales; UoL, University of Leeds; UoO, University of Otago; UoT, University of Turku.

The mean total SUS score was 80 (max 100), indicating generally high perceived usability of the VR animation. Some descriptive variation in SUS scores was observed across study sites (Figure 3), with the highest scores in the Norwegian cohort and the lowest at UoL. However, these site‐level differences should be interpreted cautiously due to uneven and small subgroup sizes. Approximately one third (43/132, 33%) reported some sort of discomfort during the VR session. No significant gender differences were observed. Free text responses included reports of a bit heavy headset, dizziness, and motion sickness. Most of the students gave the impression that the discomfort was minor and did not prevent them from completing the session.

When asked for potential improvements to the VR application, many of the respondents recommended developing some sort of interactive elements. Several suggested including subtitles or text boxes that could be opened by choice, or name tags on the different visual elements in the video. Incorporating quizzes into the application was mentioned by a few of the students. A navigation feature showing the location within the body at any given time, to avoid losing orientation, was another suggestion. Finally, many of the respondents wanted less voice‐over during the video, as it was difficult to focus on both speech and video at the same time.

### 3.2 Focus Group Interviews

The overall research aim of the focus group interviews was to explore how students perceive the usefulness of VR in learning pharmacology. Student responses were categorized into three primary themes: (1) The role of VR in integrating pharmacology with other medical disciplines; (2) striking a balance between engaging and overwhelming learning experiences; (3) in‐depth learning under the weight of assessment. The following sections elaborate on each theme.

#### 3.2.1 The Role of VR in Integrating Pharmacology With Other Medical Disciplines

This theme captures how the students perceived the integration of pharmacology with other medical subjects. Although areas like anatomy and physiology can be considered foundational for understanding pharmacology, students experienced a lack of connection between these areas of study in their program and struggled to see the bigger picture. In a description of the curriculum, respondent 7 stated that “I feel like we learn things in chunks. Hopefully, the whole picture will come together in the end.”

Students described the VR animations as helpful in visualizing connections between physiological processes in the body and see the whole picture. Pharmacological principles, allowing them to better appreciate the “big picture.” The immersive experience encouraged them to contextualize and integrate knowledge across disciplines. For instance, some highlighted the fact that they had never thought of the path that a drug follows inside the body:

I hadn't imagined that it (A/N: The tablet) ends up in many small pieces that then travel around the body. Respondent 9

Yes, that's actually true. You imagine a pill just going all the way through. Respondent 8

I don't think about the inside of organs when I imagine things. It was strange to see. I enjoyed following the medicine through the body. I haven't thought about the fact that the drug goes through the heart, for example, that it goes this way and that way and then out. You get a different perspective. You see things that you don't necessarily think about happening. Respondent 7

Not all students experienced the same novelty or “eureka moment,” even though they reported the learning experience as entertaining: “I thought it was cool to be inside the body. To see it with my own eyes. I felt that I knew what was going to happen along the way; there was a limit to how much new information I received” Respondent 12.

It was really cool. To learn you need new perspectives on things. And this is a new way of learning, a new perspective. I thought it was fun to see that journey. You kind of know what's going to happen, but as it develops further, there can be more information in it as well. It's just a start. Respondent 13

#### 3.2.2 Striking a Balance Between Engaging and Overwhelming Learning Experiences

When evaluating the VR learning experience, students explored the balance between immersion and information overload. The theme examines how students perceive and manage the tension between being highly engaged in their learning experiences and becoming overwhelmed by the volume and complexity of the academic content, or by the sensory demands of VR. The challenge of striking just the right balance between immersiveness and overload was particularly apparent in the way students discussed potential improvements. On the one hand, several students wanted more information integrated into the app including written text and interactive elements. At the same time, they were concerned about being overstimulated and that listening to the voice‐over and simultaneously watching the animations could at times be distracting, as if the different stimuli were competing for their attention.

I thought, goodness, is it me who can't do two things at the same time; listen and see. Respondent 8.

Group discussion further highlighted this tension:

Respondent 2: “It kind of becomes a bit overwhelming when you're inside the body somewhere and then there's a lot of information coming at you.” Respondent 5: “Because it's the VR part that's cool. If I want to listen to someone talking a lot, I'll go to a lecture. It's much more fun to see it.” Respondent 1: “Yes, I agree with that.”

Nevertheless, the students emphasized the importance of access to key information, and suggested alternative ways of presenting facts:

I also thought about whether some of the hard facts that were mentioned could have been omitted and replaced with the option to click on things and read about them. Respondent 4

I lost track a bit when we got to the liver. I'm not very good at English either. I forgot where I was and lost a bit of the overview. One thing I thought about is that it would have been helpful to follow along with subtitles at the same time. That way, it would be easier to keep up with things. Respondent 10

Overall, students recommended features such as live orientation, subtitles, quizzes, and gamification.

#### 3.2.3 In‐Depth Learning Under the Weight of Assessment

The data material revealed that students experienced a possible conflict between developing a deep understanding of pharmacology and meeting the demands of assessments. In the group discussions the students highlighted the importance of efficient learning methods to manage the extensive curriculum. As Respondent 7 emphasized: “It's all about time; I'm aiming for the most efficient ways to learn.” Assessment requirements were identified as a major driver of learning behavior: “Now that we have the OSCE (objective structured clinical examination), it's crucial to learn as efficiently as possible because there's so much to cover, learn the most important parts, and then accept what you can't master.”

Additionally, several of the students mentioned the time‐consuming nature of more traditional study methods such as textbook reading. This is summarized in a quote from respondent 13:

Reading books is very time‐consuming. We have an extensive curriculum to cover in a very short time. Every week there are new topics and things we need to learn. For me, reading a chapter takes 3 hours. I wish I had time to read books, but I don't.

Nevertheless, the interviews left an impression that students genuinely wanted to understand the key pharmacological principles: “I do want to become a good doctor. I want everything I do to be focused on becoming the best doctor I can be” (Respondent 13). Respondent 8 reflected on how VR helped connecting different pieces of knowledge to form a more comprehensive understanding of pharmacology: “Putting the puzzle pieces together and seeing the bigger picture becomes easier. Understanding how things are connected.”

## 4 Discussion

### 4.1 Perceived Learning Outcomes

We developed a VR animation video to enhance student engagement and understanding of core concepts by enabling students to visualize complex and otherwise “invisible” processes within the human body that are challenging to explain with speech and pictures alone. This study aimed to assess the perceived usefulness of VR in supporting learning core concepts of pharmacology. Approximately half of the respondents reported a change in their understanding of drug absorption, despite having already completed a basic pharmacology course and engaging with a non‐interactive VR experience. Many students had not previously recognized that orally administered drug molecules pass through the liver before reaching systemic circulation. This misunderstanding can cause serious medication errors, for example by incorrectly assuming that oral and intravenous doses of drugs such as morphine are equivalent. Morphine undergoes extensive first‐pass metabolism reducing its bioavailability to about 30% [38]. A clinician unfamiliar with this principle might prescribe an oral dose of morphine that is too low, resulting in insufficient pain control, or conversely, administer an “equivalent” intravenous dose without adjustment from the oral dose, leading to sedation and respiratory depression. Addressing such misconceptions is therefore essential for safe and effective prescribing.

### 4.2 Technical Usability and Suggestions for Improvements

Approximately one third of the respondents reported minor discomfort such as dizziness or nausea. This is commonly referred to as cybersickness and is a known challenge when using VR [39]. This finding may support the development of alternative technical VR solutions, that allow students greater control over the VR environment itself and the progression of the educational session. Nevertheless, the technical usability of the VR application was rated highly. The mean total SUS score of 80 is considered “good” according to Bangor et al. [35] All included sites had a total SUS score above 70, which is considered acceptable. New educational technology should be easy to learn and use [40], as technical difficulties can quickly discourage both students and educators from adopting it. The time spent becoming familiar with the technology will depend on the students' digital literacy, in addition to the design of the technology itself. In the present study, there may have been a selection bias towards students who were more interested in and familiar with new technology which in turn could have led to a higher SUS score than in a general student population. On the other hand, experienced respondents might have higher expectations regarding the technical solution and be more critical in their feedback.

The students had many suggestions for improving the VR application including interactive elements, self‐paced exploration, user decision‐making, and gamification. In their cognitive‐affective model of immersive learning (CAMIL), Makransky et al. [41] describe the general affordances of learning in VR as the sense of presence and agency. The sense of presence is the feeling of “being there,” which is determined by the degree of immersion provided by the system and the extent to which the user can modify the VR environment. The sense of agency can be described as a feeling of generating and controlling actions. Both affordances may influence factors important for learning such as motivation, self‐efficacy, self‐regulation, and cognitive load. In the VR application tested in the present study, the narrative was fixed, and it was not possible to interact within the VR world, meaning that the sense of agency was likely to be low. Future updates should aim to provide users with enhanced control over the VR environment. Instead of following a fixed narrative in a predefined VR journey, users should also be given the opportunity to actively influence the progression of the narrative.

### 4.3 Knowledge Integration

The scientific structure of medicine, divided into different disciplines and organ systems, makes it challenging for students to learn topics that require integration of knowledge from multiple areas including pharmacology. Our study indicates that VR may play an important role in supporting students' holistic understanding, by creating context and connection between medical subjects such as chemistry, anatomy, physiology, and pathology. This is exemplified by one student's response in the questionnaire: “It made me realize how connected the organs are.” Respondent 9's realization that “…the tablet ends up in many small pieces that then travel around the body” illustrates how VR can make pharmacological processes more concrete. This is consistent with research by Radianti et al., who found that immersive technologies enhance learning by providing realistic simulations that engage multiple senses, thereby supporting conceptual understanding [24].

However, the VR experience was not equally beneficial for all students. Statements such as “there was a limit to how much new information I received” (Respondent 13) suggest that while VR can increase engagement and provide new perspectives, its effectiveness may depend on students' prior knowledge and the level of detail presented. Incorporating interactive and personalized features could help address this variability by allowing content to be tailored to individual learning needs.

### 4.4 Immersiveness Versus Cognitive Overload

**FIGURE 4:**
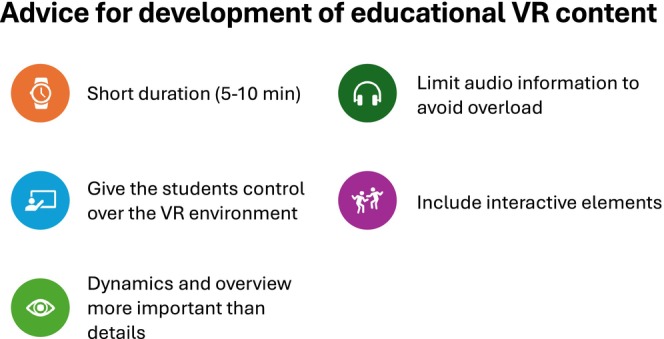
Summary of key recommendations for the development of educational VR content.

Results from both the questionnaire and focus groups indicated that many students perceived the combination of visual input and a comprehensive voice‐over in the VR video as overwhelming. The immersiveness of VR engaged the students, but they were also concerned about information overload. The use of an English voice‐over may have contributed to this experience, as a substantial proportion of the students were not native English speakers. Processing complex pharmacological content in a second language may increase cognitive load. According to Mayer's multimedia learning theory, verbal and visual information are processed through separate but capacity‐limited channels [42]. Effective learning requires filtering, organizing, and integrating inputs with prior knowledge. Utilizing both auditory and visual inputs can fully exploit this capacity, but the risk of overload must be considered, particularly when language processing itself demands additional cognitive efforts [42]. Including interactive elements and breaking content into smaller, more manageable units may help reduce cognitive load. Moreover, Mayer's principle of coherence, which advises against including superfluous information, may be particularly relevant in VR environments [42]. Future VR‐based applications may therefore benefit from minimizing unnecessary details and incorporating adaptations such as simplified language, optional subtitles, or versions in students' native languages. These strategies could enhance learning effectiveness while reducing the risk of cognitive overload. Figure 4 summarizes the key recommendations derived from this discussion.

### 4.5 The Impact of Assessment

The thematic analysis of the focus groups revealed that the participating students from UiB experienced a tension between striving for deep understanding and the need to meet assessment demands. It is well established that students tend to prioritize learning material they expect to be assessed on [43]. Consequently, the pressure to perform well in assessments can overshadow the goal of achieving deeper conceptual understanding. This is particularly relevant in medical education, where extensive curricula can lead students towards surface learning and memorization. Literature suggests that formative assessments promote deeper learning, whereas summative assessments tend to reinforce surface learning strategies [43, 44]. To maintain students' motivation and ensure that VR technology is used as an effective learning tool in the long term, its integration into teaching must be aligned with assessment practices. The initial enthusiasm for new technology can be directed towards deeper learning by introducing more formative assessments and process‐oriented tasks.

### 4.6 Limitations

Although our findings suggest that VR has the potential to enhance pharmacology education, several limitations should be acknowledged. As a pilot study, the primary aim was to gather broad feedback from a diverse cohort of students rather than to obtain a representative sample from each participating site. The heterogeneity across study sites, including differences in program structure, year of study, timing of prior pharmacology instruction, VR hardware, and language context, may have influenced both usability ratings and perceived learning outcomes. The voluntary nature of participation introduces potential selection bias, and students' prior attitudes towards VR may have influenced their experience, either through initial enthusiasm or distraction due to a novelty “wow factor” [45, 46].

Questionnaire responses may be subject to social desirability bias, and the tendency towards more positive responses at UiB may reflect participants' familiarity with the developers. To reduce acquiescence bias, we alternated positive and negative statements in Likert‐scale questions. Similar bias could also have influenced the students in the focus groups facilitated by T.T.S. and J.A.B.

We also experienced some participant dropout from the focus group interviews, which may have resulted in the loss of important perspectives. In addition, the qualitative data were generated at a single site within one disciplinary context, potentially limiting the transferability of the findings due to local curricular structures and assessment practices. Although two of the identified themes are broadly consistent with existing literature [41, 47, 48], the theme concerning the role of VR in integrating pharmacology with other medical disciplines remains underexplored.

Finally, we did not explore whether students' academic performance influenced their perceptions of VR, an area which should be of interest for future research.

## 5 Conclusions

In the present study, we found that a 360° VR video can enhance student engagement and motivation in pharmacology learning. To maximize its benefits, VR applications should be designed to address specific gaps in students' understanding, helping to correct misconceptions and potentially reduce medication errors due to insufficient pharmacological knowledge. The design of VR applications must carefully balance immersiveness with cognitive overload while also aligning with the broader curriculum, including assessment design, to ensure integration into students' learning experiences. Future research should explore how VR can be tailored to individual needs, using new technologies, including AI, and evaluate its long‐term impact on knowledge retention and application in clinical practice.

## Author Contributions

J.A.B., T.T.S., M.K., M.C.S., and P.W. conceived the study and developed the overall study design; J.A.B., T.T.S., and the local site investigators coordinated the implementation of the VR intervention and data collection across the participating institutions; J.A.B. led the quantitative analysis, with input from the local site investigators (T.T.S., J.L., W.B., S.G., D.R., U.P., D.M., S.S.); M.K. led the qualitative analysis, with contributions from J.A.B., T.T.S. and T.E.M.; J.A.B. drafted the first version of the manuscript. All authors contributed to interpretation of the findings, critically revised the manuscript for important intellectual content, and approved the final version for submission.

## Funding

This study was supported by University of Bergen, Digitalisation funds.

## Conflicts of Interest

The authors declare no conflicts of interest.

## Supporting information


**Data S1:** prp270294‐sup‐0001‐DataS1.pdf.

**Data S2:** prp270294‐sup‐0002‐DataS2.pdf.

**Data S3:** prp270294‐sup‐0003‐DataS3.pdf.

## Data Availability

The data generated and analysed during the current study are not publicly available due to ethical and privacy considerations, as they include questionnaire responses and focus group data from student participants that may not be fully anonymisable. Reasonable requests for access to aggregated or de identified data may be directed to the corresponding author, subject to institutional and ethical approvals.
