# Supplementary material for: Student Perceptions of a Virtual Reality Animation for Teaching Absorption and Bioavailability in Pharmacology: A Mixed Methods Evaluation
Source: Pharmacol Res Perspect. 2026 Jul 1;14(4):e70294. doi: 10.1002/prp2.70294 (PMC13323160; doi:10.1002/prp2.70294)
Supplement: Supplementary file 1 — Data S1: prp270294‐sup‐0001‐DataS1.pdf. [file PRP2-14-e70294-s003.pdf]

Interview guide “Pharmacology education in VR”

Translated from Norwegian

Introduction.

You have all now seen a pilot animation in VR that describes the journey of drug molecules from being swallowed as a tablet to reaching the heart.

The purpose of today's discussion is to delve into your thoughts and feelings and to elicit reflections that can shed light on topics related to this type of educational material. The aim of conducting a group interview is to create a dynamic and an interaction that allows each of you to generate new ideas to build upon. We encourage you to be open and honest in the conversation and would like to emphasize that there are no "wrong" answers.

Before we start, we would like to remind you that all information shared during the interview will be treated confidentially, and any published material will be anonymized. We will be recording the conversation, and the recording will be deleted after transcription.

We will continue until we are out of topics.

We want to hear your thoughts on the product as it currently stands and would first like to talk about learning and the use of educational materials.

1. What do you usually use as educational materials in the medical program?

2. Are there specific factors that influence whether an educational material is used or not?

We have now talked about the use of various educational materials.

3. How do you think VR is suitable as an educational tool in the medical program?

4. If it becomes available, how will you use it?

5. What changes are needed for the pilot animations to be as useful as possible as an educational tool for you?

It is well known and completely understandable that students are concerned with what material is relevant for exams.

6. Do you think the animations you have seen today are exam-relevant material?

7. How could VR potentially be used in an exam context?
