# Supplementary material for: Student Perceptions of a Virtual Reality Animation for Teaching Absorption and Bioavailability in Pharmacology: A Mixed Methods Evaluation
Source: Pharmacol Res Perspect. 2026 Jul 1;14(4):e70294. doi: 10.1002/prp2.70294 (PMC13323160; doi:10.1002/prp2.70294)
Supplement: Supplementary file 2 — Data S2: prp270294‐sup‐0002‐DataS2.pdf. [file PRP2-14-e70294-s001.pdf]

VR pharmacology: Feedback Survey

The purpose of this survey is to gather feedback from pharmacology students who have used the prototype VR application designed for teaching pharmacology concepts. The survey focuses on the usability of the application, its impact on motivation and engagement, and any potential areas for improvements. You will need to answer each question to complete the questionnaire.
The quiz will take around 10 minutes to complete. Participation in the study is completely voluntary. Your answers will be anonymous.

The first part is about the usability of the application you just tested. Please respond based on how much you agree with the statements.

I think that I would like to use this VR application frequently.

Strongly Disagree	Disagree	Neither Agree nor Disagree	Agree	Strongly Agree
☐	☐	☐	☐	☐

I found the VR application unnecessarily complex.

Strongly Disagree	Disagree	Neither Agree nor Disagree	Agree	Strongly Agree
☐	☐	☐	☐	☐

I thought the VR application was easy to use.

Strongly Disagree	Disagree	Neither Agree nor Disagree	Agree	Strongly Agree
☐	☐	☐	☐	☐

I think that I would need the support of a technical person to be able to use the VR application.

Strongly Disagree	Disagree	Neither Agree nor Disagree	Agree	Strongly Agree
☐	☐	☐	☐	☐

I found the various functions in the VR application were well integrated.

Strongly Disagree	Disagree	Neither Agree nor Disagree	Agree	Strongly Agree
☐	☐	☐	☐	☐

I thought there was too much inconsistency in the VR application.

Strongly Disagree Disagree Neither Agree nor Disagree Agree Strongly Agree

☐ ☐ ☐ ☐ ☐

I would imagine that most people would learn to use the VR application very quickly.

Strongly Disagree Disagree Neither Agree nor Disagree Agree Strongly Agree

☐ ☐ ☐ ☐ ☐

I found the VR application very awkward to use.

Strongly Disagree Disagree Neither Agree nor Disagree Agree Strongly Agree

☐ ☐ ☐ ☐ ☐

I felt very confident using the VR application.

Strongly Disagree Disagree Neither Agree nor Disagree Agree Strongly Agree

☐ ☐ ☐ ☐ ☐

I needed to learn a lot of things before I could get going with this VR application.

Strongly Disagree Disagree Neither Agree nor Disagree Agree Strongly Agree

☐ ☐ ☐ ☐ ☐

Watching the VR application made me think differently about drug absorption.

Strongly Disagree Disagree Neither Agree nor Disagree Agree Strongly Agree

☐ ☐ ☐ ☐ ☐

Watching the VR application did not in any way conflict with my understanding of drug absorption.

Strongly Disagree Disagree Neither Agree nor Disagree Agree Strongly Agree

☐ ☐ ☐ ☐ ☐

We would now like you to answer some questions in free text, to help us further develop the VR application.

Which part of the VR application did you find the most interesting or engaging, and why?

Did you experience any technical challenges or limitations with the VR application?

What changes or new features would you recommend for the VR application to enhance motivation and engagement while learning pharmacology?

Did the VR animation change your understanding of drug absorption? If yes, please describe briefly.

❑ No

❑ Yes_________

Did you experience any discomfort when using the VR application? If yes, please describe briefly.

❑ No

❑ Yes_________

Following are 10 statements about the use of VR technology for learning pharmacology. Please respond based on how much you agree with the statement.

Using VR technology in pharmacology education can make learning more engaging.

Strongly Disagree	Disagree	Neither Agree nor Disagree	Agree	Strongly Agree
❑	❑	❑	❑	❑

VR technology does not provide any learning benefits compared to traditional teaching methods such as lectures.

Strongly Disagree	Disagree	Neither Agree nor Disagree	Agree	Strongly Agree
❑	❑	❑	❑	❑

VR technology can help me understand complex pharmacological concepts more easily.

Strongly Disagree	Disagree	Neither Agree nor Disagree	Agree	Strongly Agree
❑	❑	❑	❑	❑

Using VR technology in pharmacology education is a waste of resources.

Strongly Disagree	Disagree	Neither Agree nor Disagree	Agree	Strongly Agree
❑	❑	❑	❑	❑

Utilizing VR technology in pharmacology education can improve my motivation to learn.

Strongly Disagree Disagree Neither Agree nor Disagree Agree Strongly Agree

❑ ❑ ❑ ❑ ❑

The use of VR technology in pharmacology education is distracting.

Strongly Disagree Disagree Neither Agree nor Disagree Agree Strongly Agree

❑ ❑ ❑ ❑ ❑

VR technology provides a unique approach to learning pharmacology.

Strongly Disagree Disagree Neither Agree nor Disagree Agree Strongly Agree

❑ ❑ ❑ ❑ ❑

The benefits of using VR technology in my field of study do not outweigh the challenges and limitations.

Strongly Disagree Disagree Neither Agree nor Disagree Agree Strongly Agree

❑ ❑ ❑ ❑ ❑

The use of VR technology will make it easier for me to retain pharmacological knowledge.

Strongly Disagree Disagree Neither Agree nor Disagree Agree Strongly Agree

❑ ❑ ❑ ❑ ❑

The implementation of VR technology in pharmacology education adds unnecessary complexity.

Strongly Disagree Disagree Neither Agree nor Disagree Agree Strongly Agree

❑ ❑ ❑ ❑ ❑

Finally, a few questions about you

Please indicate your gender.

Woman/female Man/male Non-binary Prefer not say I use a different term

❑ ❑ ❑ ❑ ❑ ____________

At which university are you currently enrolled?

❑ University of Bergen
❑ University of Turku
❑ University of Leeds
❑ Monash University
❑ University of New South Wales
❑ University of Otago
❑ Other (please name)__________

In what year of program are you enrolled?

1 2 3 4 5 6

❑ ❑ ❑ ❑ ❑ ❑

In what program are you enrolled?

❑ Medicine

- [ ] Pharmacy
- [ ] Pharmaceutical science
- [ ] Biomedicine
- [ ] Other (please name):

How many times have you tried VR before?

Never	1 time	2-5 times	More than 5 times
☐	☐	☐	☐
