# Supplementary material for: Student Perceptions of a Virtual Reality Animation for Teaching Absorption and Bioavailability in Pharmacology: A Mixed Methods Evaluation
Source: Pharmacol Res Perspect. 2026 Jul 1;14(4):e70294. doi: 10.1002/prp2.70294 (PMC13323160; doi:10.1002/prp2.70294)
Supplement: Supplementary file 3 — Data S3: prp270294‐sup‐0003‐DataS3.pdf. [file PRP2-14-e70294-s002.pdf]

Survey items	Strongly disagree n (%)*	Disagree	Neither agree nor disagree	Agree	Strongly agree	n
**Using VR technology in pharmacology education can make learning more engaging.**	1 (1)	0	2 (2)	43 (33)	86 (65)	132
**VR technology does not provide any learning benefits compared to traditional teaching methods such as lectures.**	51 (39)	63 (48)	12 (9)	5 (4)	1 (1)	132
**VR technology can help me understand complex pharmacological concepts more easily.**	0	4 (3)	8 (6)	55 (42)	65 (49)	132
**Using VR technology in pharmacology education is a waste of resources.**	55 (42)	53 (40)	13 (10)	9 (7)	2 (2)	132
**Utilizing VR technology in pharmacology education can improve my motivation to learn.**	0	5 (4)	5 (4)	64 (48)	58 (44)	132
**The use of VR technology in pharmacology education is distracting.**	51 (39)	59 (45)	12 (9)	8 (6)	2 (2)	132
**VR technology provides a unique approach to learning pharmacology.**	0	1 (1)	1 (1)	48 (36)	82 (62)	132
**The benefits of using VR technology in my field of study do not outweigh the challenges and limitations.**	26 (20)	47 (36)	27 (20)	24 (18)	8 (6)	132
**The use of VR technology will make it easier for me to retain pharmacological knowledge.**	2 (2)	5 (4)	7 (5)	62 (47)	56 (42)	132
**The implementation of VR technology in pharmacology education adds unnecessary complexity.**	45 (34)	63 (48)	11 (8)	12 (9)	1 (1)	132
**Watching the VR application made me think differently about drug absorption.**	1 (1)	20 (15)	30 (23)	60 (45)	22 (17)	133
**Watching the VR application did not in any way conflict with my understanding of drug absorption.**	3 (2)	6 (5)	20 (15)	64 (48)	40 (48)	133

S3.

*Percentages may not sum to 100 due to rounding. Totals may differ slightly across items because one participant did not respond to some of the questions’
